# Microbial diversity at remediated former gold and copper mines and the metal tolerance of indigenous microbial strains

**DOI:** 10.1128/aem.02555-24

**Published:** 2025-08-29

**Authors:** Vira Velianyk, Martin Palusak, Nhung Huynh Anh Nguyen, Jakub Riha, Alena Sevcu, Miroslav Cernik, Veronika Hlavackova

**Affiliations:** 1 Department of Applied Biology, Institute for Nanomaterials, Advanced Technologies and Innovation, Technical University of Liberec, Liberec, Czech Republic; 2 Faculty of Science, Humanities and Education, Technical University of Liberec, Liberec, Czech Republic; Colorado School of Mines, Golden, Colorado, USA

**Keywords:** acid mine drainage, tailing site, bioremediation, metal-tolerant microorganisms, polymetallic ore

## Abstract

Metamorphic Devonian rocks in the northern Moravian Jeseník district (Czech Republic) contain non-vein polymetallic, copper, and gold deposits. Sulfide leaching following previous mining activities has caused significant chemical and physical alterations in the local environment, resulting in biological process shifts. Here, we present a detailed microbiological survey of the Zlaté Hory mine and its adjacent features, including tailings, sediments, and treated mine water. In addition, we isolated metal-tolerant bacteria and fungi as potential candidates for the bioremediation of mine wastewater. Microbial community analysis revealed differences driven by environmental conditions. Acidotolerant consortia, for example, *Gallionella* sp. and *Ferrovum* sp., were mainly detected in the acidic shaft outflow (pH 3.07, conductivity 1,351 µS.cm^−1^). Subsequent treatment and pH neutralization led to the presence of metal-tolerant heterotrophs, including fungi, despite elevated heavy metal concentrations. Notably, the highest microbial diversity was observed in drainage water with low metal content. These findings suggest that physicochemical factors, such as pH and metal/metalloid concentration, play a pivotal role in shaping environmental microbiomes and influencing community composition. Furthermore, while biogeochemical processes (e.g., Fe(II) oxidation, sulfide precipitation, metal immobilization) may already be contributing to natural mine water remediation, unfavorable equilibria in the iron cycle caused by acidification could be compensated for through bioremediation using beneficial microorganisms, such as *Variovorax* or *Arthrobacter* isolates.

**IMPORTANCE:**

Microorganisms play a crucial role in the biogeochemical cycles of elements, for example, carbon and sulfur, and metals. As ubiquitous ecosystem components, they have a significant influence on most processes on Earth. Investigating microbial diversity is essential for understanding these processes, particularly in extreme environments such as mining sites. Microorganisms from mining sites often develop resistance to harsh conditions, including high concentrations of heavy metals and acidity. In addition, certain microbes can metabolize or transform toxic substances, contributing to the remediation of other contaminated environments. As mining activities persist or legacy sites degrade, microbial data become invaluable for predicting long-term environmental impacts and informing sustainable management practices.

## INTRODUCTION

Ever since the Bronze Age, mining has been a critical industry supporting human development, initially by providing essential metals and minerals for various crafts and now serving advanced industrial sectors such as electronics, energy production, construction, and pharmaceutical production. The historical extraction of rich vein metal sources, followed by extensive exploitation of dispersed minerals in ores, has led to a general depletion of critical element resources (1). In addition, modern mining practices, such as open chamber technology, have led to significant ecological impacts, including the continuous weathering of sulfidic ores and generation of acid mine drainage (AMD), which facilitates the mobilization of metals and metalloids and poses a serious threat to water quality and ecosystems generally. AMD treatment is essential to mitigate the harmful effects of toxic contaminants in groundwater; however, the AMD treatment process also produces toxic precipitates that must typically be stored at tailing sites, where they pose ongoing environmental risk. The combination of resource depletion and accumulation of concentrated toxic waste necessitates alternative approaches to secure valuable raw materials.

To address the demand for sustainable mining, so-called “zero-waste” mining strategies are being developed that enhance mining efficiency while minimizing waste production, its environmental deposition, and its associated ecological footprint (2). Such strategies usually involve the direct recycling of mining by-products, including wastewater, red mud, tailings, and metallurgical slag (3, 4).

A promising low-cost alternative strategy for recovering critical elements, which is both environmentally friendly and more sustainable, involves the use of diverse biota, including bacteria, fungi, and plants. Many of these organisms can tolerate the toxic burden of contaminated sediments, soils, and waters by precipitating out heavy metals (5–9). Consequently, they can be effectively utilized for the bioremediation of polluted areas (10–12). Microbial potential to tolerate and transform heavy metals has recently been studied at several former mining sites in the Czech Republic.

**Fig 1:**
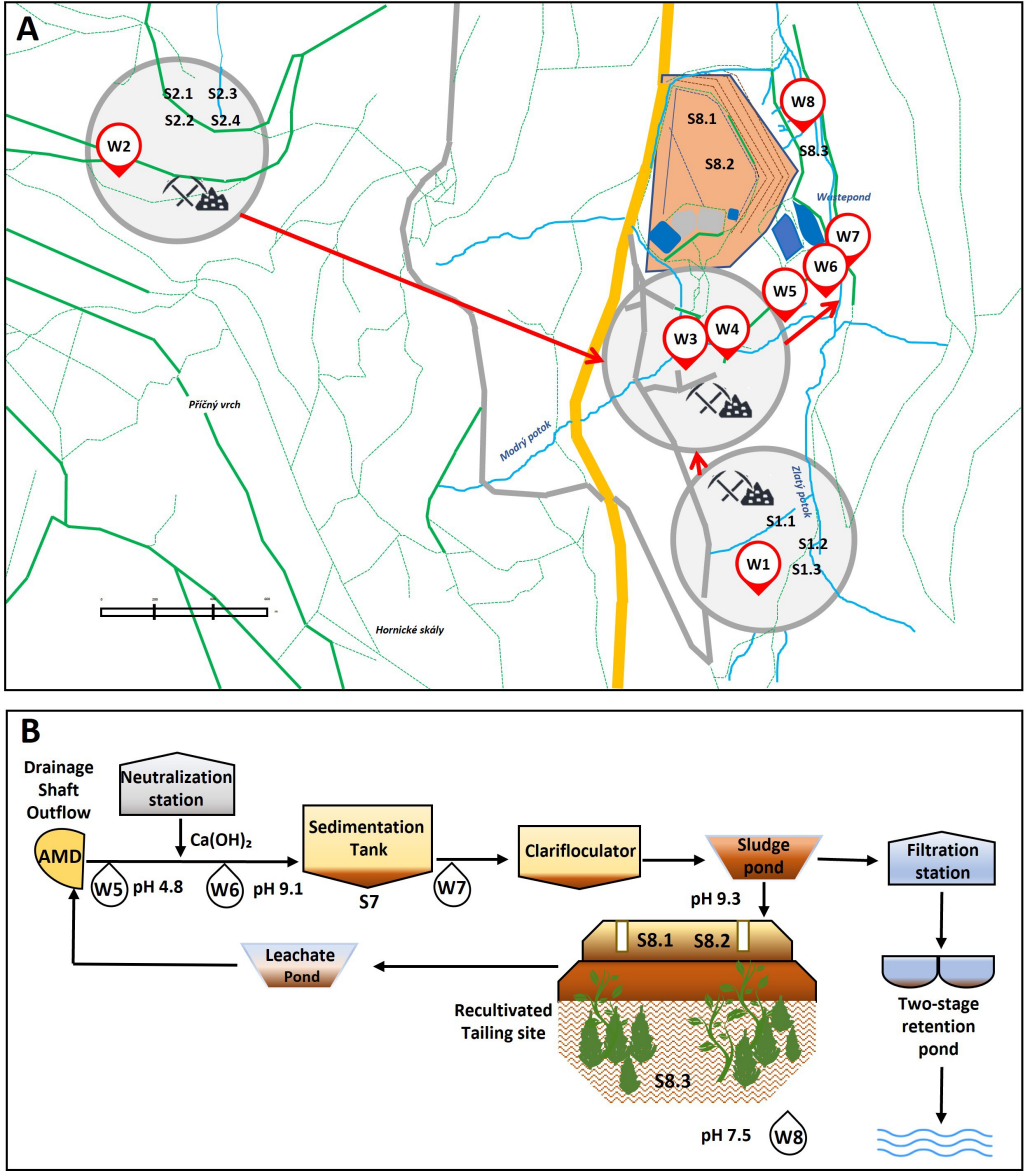
Schematic overview of the two campaigns of sampling in the former Zlaté Hory mining site (**A**) and water treatment plant (**B**).

To evaluate the bioremediation potential of autochthonous microbial communities at former mining sites, this study first characterized the geochemistry, structure, and composition of the microbial community in mine water, focusing on both bacteria and fungi. The use of a comprehensive sampling approach, which included water samples, solid samples, sludges, and sediments (Fig. 1; see Fig. S1 and S2 at https://doi.org/10.6084/m9.figshare.29616554), enabled the investigation of changes in microbial diversity from underground to surface areas. In addition, it facilitated the analysis of microbial community shifts in response to adjacent AMD treatment as well as the impact of concentrated toxic loads on the microbial community in tailings and sediments. By exploring the relationship between physicochemical parameters and microbial diversity, this study provides insights into how microbes adapt to extreme and dynamic environments, contributing to the development of novel strategies for bioremediation of such settings. As a second objective, metal-tolerant bacteria capable of withstanding high environmental toxicity were then cultivated and investigated as promising candidates for bioremediation.

## RESULTS AND DISCUSSION

### Geochemical characterization of mine waters

The geochemical characterization of mine waters from Zlaté Hory (W1 and W4) revealed high specific conductivity at 1,351 µS.cm^−1^ and 1,221 µS.cm^−1^, respectively, and reduction potential, at 237 mV and 155 mV, respectively (Table 1). Nevertheless, W1 water had a distinctly acidic pH (3.07), while W4 water was neutral (7.40). The highest elevated concentrations of sulfates were detected in W1 water, at 1,174 mg.L^−1^ (see Table S1 at https://doi.org/10.6084/m9.figshare.29616554). These findings align with the general chemistry of mine waters and reflect the acidic leaching of sulfide minerals and production of sulfuric acid, with low pH conditions resulting in further extensive mineralization (13, 14).

**TABLE 1:** Physicochemical parameters of water samples used in this study

Sample	T^*a*^ [°C]	pH	O_2_^*b*^ [mg.L^−1^]	ORP^*c*^ [mV]	C^*d*^ [µS.cm^−1^]
Underground					
W1	7.00	3.07	9.31	237	1,351
W2	8.10	4.60	9.59	376	403
W3	7.60	7.70	10.8	282	325
W4	8.80	7.40	10.6	155	1,221
Surface					
W5	8.60	4.80	10.3	330	568
W6	9.20	9.10	10.0	105	660
W7	10.7	9.30	8.50	95.0	670
W8	10.9	7.50	10.20	−37.0	218

^*a*^ T = temperature.

^*b*^ O_2_ = dissolved oxygen.

^*c*^ ORP = oxidation-reduction potential.

^*d*^ C = conductivity.

As at other mining sites, sample W1 exhibited elevated levels of dissolved aluminum, arsenic, copper, iron, manganese, and zinc (15–19), the results revealing significant variation in concentrations between samples (Table S1). Notably, the acidic W1 water sample and the water from the sedimentation tank W7 both contained the highest total levels of metals such as copper, iron, manganese, and aluminum. Remarkably, the concentration of silver in sample W7 was measured at 2.28 mg.L^−1^, confirming enrichment in the sedimentation tank (Table S1). Similarly, zinc concentrations were exceptionally high in W7, with notable increases also observed in sample W2.

Regarding anions, sulfates exhibited the highest concentrations in the acidic water sample W1 and the tailing site water sample W8 (Table S1). These findings underscore the influence of water treatment on the distribution of metals and anions and highlight the critical role of acidity neutralization and the concentration of elements during sedimentation.

According to the AMD classification of Opitz and Timms (20), the relatively low concentrations of iron, at 34.4 mg.L^−1^ and 13.3 mg.L^−1^, respectively, and aluminum, at 26.5 mg.L^−1^ and 10 mg.L^−1^, respectively, place samples W1 and W2 into Class II AMD, described as partially oxidized water. Similarly, the outflow sample W5, with sulfate concentrations close to 500 mg.L^−1^ and an acidic pH of 4.8, also falls into the partially oxidized AMD category.

By contrast, samples W3 and W4 exhibited very low or undetectable concentrations of iron and aluminum, thus placing them into Class III (neutral) or Class IV (oxidized and neutralized) AMD (20). Samples W6 and W7, representing treated alkaline waters, showed distinct characteristics, with water from the sedimentation tank (W7) exhibiting elevated concentrations of aluminum, copper, iron, and zinc. Lastly, water emerging beneath the sediments displayed circumneutral pH, and while most hazardous heavy metals were depleted (Table S1), concentrations of iron and manganese remained comparable to those in acidic mine water (W1). Nevertheless, the high sulfate concentration (797 mg.L^−1^) and low levels of aluminum and iron (Table S1) would still classify this water as neutral Class III AMD (20).

The toxicity of metals can be classified based on their influence on living organisms and human health. Some metals, such as zinc, iron, and copper, are essential for the biological functions of living cells and are usually necessary for correct enzyme activity, for example, by completing enzyme structures, while others have catalytic and/or regulatory functions. On the other hand, metals, such as cadmium and lead, exhibit high toxicity due to their strong affinity for binding with biological molecules, primarily through sulfur, resulting in detrimental health effects even at low exposure levels (21). Concentrations of these two heavy metals in outflow water W8 were below the detection limit 0.01 mg.L^−1^, that is, well below the maximum recommended World Health Organization (WHO) limits for lead and cadmium of 0.01 and 0.003 mg.L^−1^, respectively.

The concentration of zinc in surface waters is usually below 10 µg.L^−1^, and ranges between 10 and 40 µg.L^−1^ in groundwater (22), while WHO guideline for zinc in drinking water sets a maximum allowable concentration of 5 mg.L^−1^ (23). In our study, the zinc concentration in the outflow creek water W8 was 0.3 mg.L^−1^, which is still well within the recommended WHO limit. While zinc at this concentration is unlikely to pose any acute health risk, its long-term effects on aquatic organisms may depend on its bioaccumulation potential.

By contrast, the concentration of iron in creek water W8 exceeded the WHO guideline value for drinking water (0.3 mg.L^−1^) by more than two orders of magnitude (24) (Table S1). Though the observed concentration is not considered harmful to mammals, based on an acute toxic dose in mammals (measured in rats) ranging from 800 to 2,000 mg.kg^−1^ of body weight (24), aquatic organisms may be more sensitive. While iron does not readily bioaccumulate, it can contribute to eutrophication and reduce oxygen availability, posing a significant ecological risk.

Copper, an essential trace element, has a WHO guideline value of 2 mg.L^−1^ for drinking water and a recommended daily intake of 900 µg for adult humans (25). Thus, the copper concentration detected in the outflow creek W8 (0.03 mg.L^−1^) can be considered safe for human consumption. However, a potential risk to aquatic ecosystems cannot be excluded due to copper’s known toxicity to aquatic species even at low concentrations, and its capacity for bioaccumulation (26–28).

### Geochemical characterization of solid samples

Solid samples, including sinters, stalks, sludges, and sediments, exhibited distinct chemical properties. Notably, sinter S1.1, collected from the ZH-South underground mine, exhibited a distinct blue color. As previously reported (13), these samples contained significant concentrations of copper (45.6 mg.kg^−1^) and other toxic heavy metals, including aluminum, arsenic, cadmium, manganese, and nickel, as previously reported. By contrast, sinter S1.2 from the same location had a white color and was predominantly made up of zinc, with a concentration of 150 mg.kg^−1^ (see Table S2 at https://doi.org/10.6084/m9.figshare.29616554).

Sinter samples S2.1 and S2.2 from ZH-West were characterized by elevated concentrations of iron, as evidenced by their distinct brown coloration. Notably, S2.2 exhibited a high metal(loid) concentration, compared to S2.1, with values in the following order: silver = vanadium < arsenic < cobalt < aluminum < lead << zinc << manganese << iron. Sludge sample S7, collected from a sedimentation tank after addition of lime milk, contained high amounts of aluminum (53,437 mg.kg^−1^), zinc (49,944 mg.kg^−1^), iron (40,843 mg.kg^−1^), copper (34,077 mg.kg^−1^), and manganese (19,284 mg.kg^−1^), precipitated after neutralization and aeration (Table S2).

Samples from the tailing site (S8.1 and S8.2) are hard to describe in terms of chemistry as the tailing site itself is composed of diverse solid waste, collected over several decades, from underground processing, and more recently from mine water treatment. The tailing site has been subjected to long-term chemical leaching processes and retains significant levels of toxic elements (Table S2). Apart from high concentrations of iron, ranging from 39,646 to 66,703 mg.kg^−1^, concentrations of heavy metals followed an order: chromium <cadmium ~ cobalt ~nickel < arsenic <manganese < lead ~copper < zinc <aluminum (Table S2). Sediment sample S8.3, collected from beneath the tailing site, was characterized by exceedingly high concentrations of iron (454,464 mg.kg^−1^), followed by aluminum, zinc, lead, and manganese. Interestingly, arsenic, along with other toxic heavy metals, such as cadmium, cobalt, copper, and chromium, was not detected in the sediment (Table S2), suggesting their retention in the tailing site. The long-term retention and concentration of heavy metals in tailings poses significant environmental risks, with tailing eros in particular, leading to the release of toxic metals into adjacent ecosystems, negatively affecting both soil and water quality (29, 30).

Arsenic is commonly found at high concentrations in tailings originating from metal mining activities due to the leaching of arsenopyrite, with concentrations depending on geographical localization, ore extraction technology, etc. For example, in the Shimen realgar mine in China, arsenic concentrations in tailings were reported to be 6.29 mg.kg^−1^ (31). While here we report 10–25 times higher concentrations in Zlaté Hory tailings (Table S2), tailings in a metal mine in Korea contained arsenic in a much higher concentration of 67,336 mg.kg^−1^ (32). The retention of arsenic in tailings is influenced both by its chemical speciation and the presence of minerals that can adsorb arsenic. Arsenic can be retained through adsorption onto clays and metal oxides, particularly those of iron, aluminum, and manganese (33), which is in accord with our chemical analysis data. As above, the long-term retention and concentration of arsenic in tailings poses significant environmental risks, with erosion in particular leading to its release into adjacent ecosystems, affecting soil and water quality. It has been shown that arsenic bound to sulfide minerals, such as arsenopyrite, is more susceptible to alteration and mobilization than when bound to silicate minerals (34). It has also been reported that arsenic mobility and leaching from zinc-lead-rich tailings is significantly influenced by the pH of leaching water, the contact time of the tailings with water, the particle size of the tailings, and the solid-to-liquid ratio (35). These findings highlight the need for ongoing monitoring and remediation efforts at mining sites to mitigate the risks associated with arsenic contamination.

### Bacterial biodiversity and community composition

Bacterial community size was assessed through amplification of the 16S rRNA gene region. After analysis, a total of 592,799 and 529,712 bacterial ASVs were identified in water and solid samples, respectively. Determined by qPCR (see Suppl. Methods at https://doi.org/10.6084/m9.figshare.29616554), however, the relative quantity of bacteria in each sample proved to be dependent on immediate treatment, pH, and oxygen level (see Table S3 at https://doi.org/10.6084/m9.figshare.29616554).

**Fig 2:**
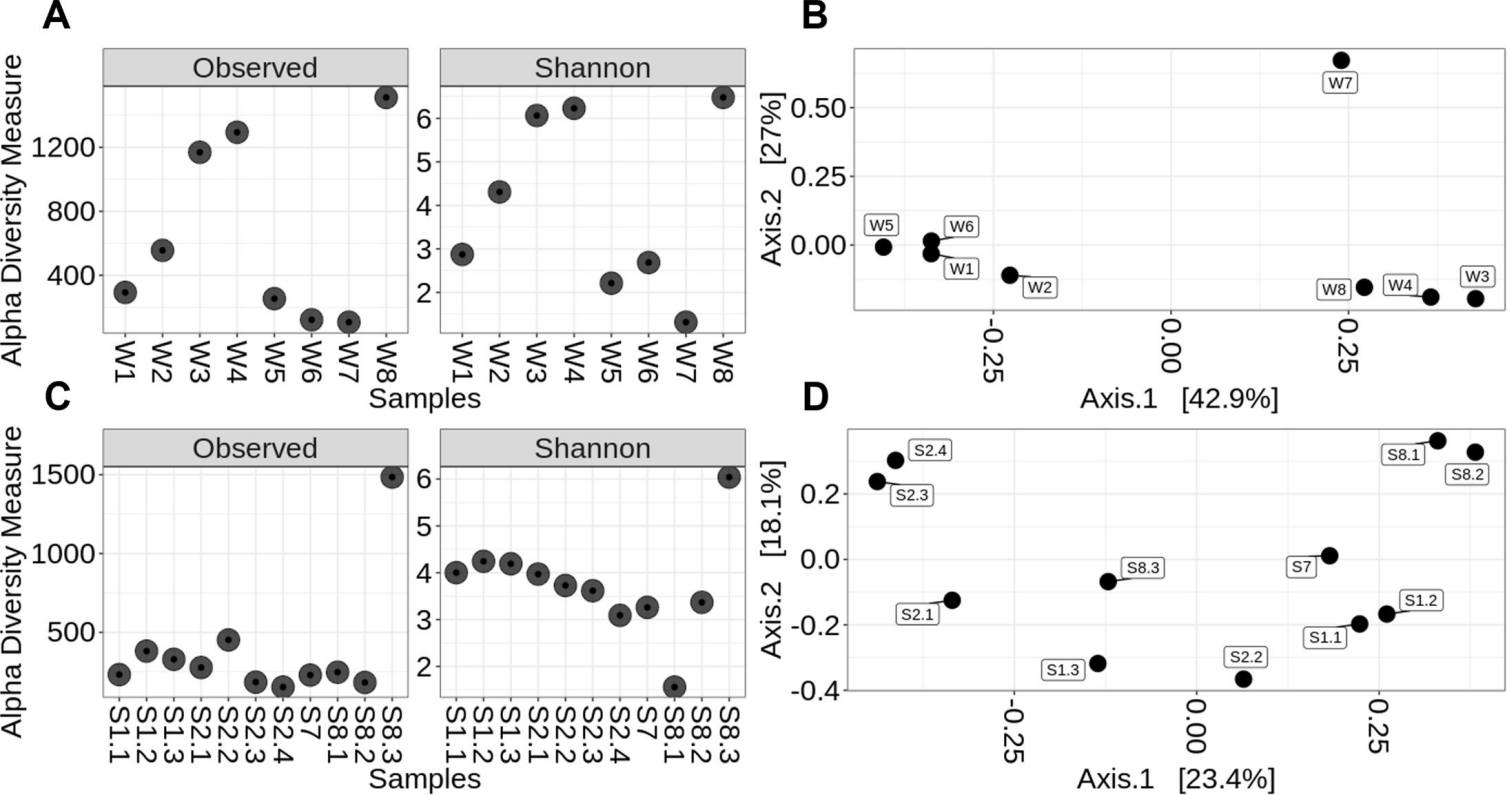
Alpha diversity (**A and C**) and PCoA (**B and D**) of bacterial communities in water (**A and B**) and solid (**C and D**) samples, based on data from sequencing analysis using the 16S rRNA primer targeting bacteria.

Based on 16S rRNA gene sequencing, the diversity of water samples could be categorized into three groups (Fig. 2A). The first group included samples W1, W5, W6, and W7, which exhibited the lowest biodiversity across all evaluated criteria. The second group comprised samples W3, W4, and W8, collected from ZH-East, with Shannon index values of 6.23, 6.07, and 6.49, respectively, representing the highest diversity among all water samples. Moderate diversity was exhibited by sample W2, obtained from an underground gold mine, with a Shannon index value of 4.34.

Clustering patterns among water samples showed a clear correlation with water type and physicochemical parameters, such as pH (Fig. 2B). Notably, the PCoA results closely mirrored those observed for alpha-diversity. At the genus level, the first cluster included samples W5 and W6, alongside the representative underground water samples W1 and W2 (Fig. 2B). The second group of closely related water samples included samples W3, W4, and W8. Sample W7 differed significantly at the genus level and exhibited the lowest alpha-diversity, suggesting the presence of just a few highly abundant genera (Fig. 2).

Bacterial community diversity in solid samples appeared uniform and low except for sample S8.3 (Fig. 2C, left). However, using Shannon diversity, a different trend was observed, with sample S8.1 exhibiting the lowest diversity, all other samples except S8.3 exhibiting moderately higher diversity, and sample S8.3 displaying the highest diversity of all samples. Most samples (S1.1, S1.2, S1.3, S2.1, S2.2, S2.3, S2.4, S7, and S8.2) exhibited Shannon index values ranging between 3 and 5, with sludge sample S8.1 having the lowest value and sediment sample S8.3 achieving the highest value (6.05; Fig. 2C, right). The discrepancies between observed diversity and Shannon index values are likely to have been caused by the presence of a few dominant genera within certain samples.

Clustering patterns for solid samples were less consistent than for water samples, with similarities linked primarily to sample origin. Interestingly, based on genus composition similarities, sediment sample S8.3, originating from the tailing site, was positioned between samples from underground locations 1 and 2 (Fig. 2D).

The former mining site at Zlaté Hory harbored a diverse bacterial community composed primarily of species from the phylum *Pseudomonadota* (*Proteobacteria*), which accounted for up to 96% of the bacterial population. This was followed by *Patescibacteria* (up to 30%), *Verrucomicrobiota* (up to 10%), and *Acidobacteriota* (up to 10%), with other bacterial phyla being less abundant (see Fig. S3 at https://doi.org/10.6084/m9.figshare.29616554). *Pseudomonadota* and *Acidobacteria* are commonly associated with mine environments, suggesting a considerable tolerance to heavy metal stress (10, 36–39)

Microbial abundance in water samples was similar overall, except for the sample undergoing neutralization treatment, where microbial diversity was distinctly different. Across all water samples, 10 bacterial genera were detected at relative abundances > 1%. Overall, a few dominant genera appeared to outcompete less adapted genera, reflecting selective pressures imposed by the extreme physicochemical conditions.

**Fig 3:**
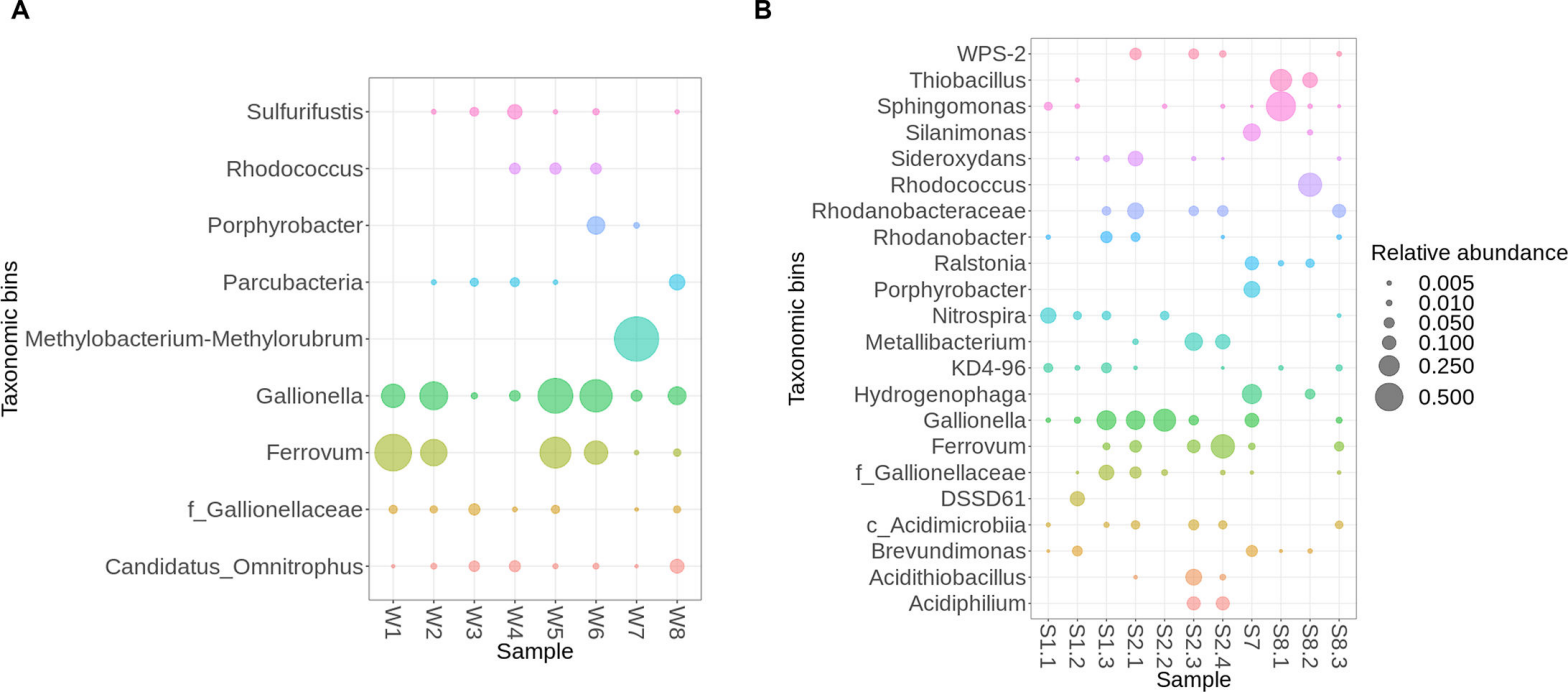
Relative abundance of bacterial phyla based on sequencing of the 16S rRNA gene in water (**A**) and solid (**B**) samples.

Acidic water from the gold mine (W1; see Fig. S1 at https://doi.org/10.6084/m9.figshare.29616554) exhibited elevated concentrations of copper, manganese, aluminum, and sulfates and had the lowest pH (3.07). This environment supported acidophilic and acidotolerant microbial consortia, with *Ferrovum* (53%) and *Gallionella* (20%) dominating the community (Fig. 3A). The presence of FeOB in underground mine waters has previously been reported (13), suggesting stability in the mine water system. In all water samples, iron concentrations ≥ 4 mg·L^−1^ were associated with a significantly increased abundance of FeOB. Other microbial taxa were suppressed by these dominant species, with relative abundances not exceeding 1%.

By contrast, water samples W3 and W4 from the ZH-East mine (Fig. S1), which had near-neutral pH, exhibited a reduced total microbial abundance compared to the acidic samples W1 and W2. Based on the origin and distinct geochemistry of samples W3 and W4, characterized by low concentrations of iron and other metals and metalloids, it is likely that there are no further oxidizable mineralization remains along the flow path from the former gold/copper mine. The lack of ferric ions results in diminished acidification and a pH shift toward neutrality, thereby altering the structure of the microbial community (Fig. 3A). The presence of sulfates in sample W4 suggests ongoing sulfur cycling, likely driven by sulfur-oxidizing bacteria such as *Sulfurifustis* (6%), which is capable of oxidizing thiosulfate, tetrathionate, and elemental sulfur, and typically thrives under circumneutral conditions (40, 41). In sample W4, the reduced presence of *Gallionella* (3%) reflects this lowered iron concentration (0.59 mg·L^−1^; Table S1; Fig. 3A).

At our mine site, all underground waters eventually converge into a combined stream that reaches the surface treatment area (Fig. 1), suggesting that microbiology of the mixed waters will be determined by the most prevalent genera. The water entering the treatment plant (W5; Fig. S1) proved to be acidic (pH 4.8), with an iron content of 4.55 mg mg·L^−1^, and consequently had a community dominated by *Gallionella* (48%) and *Ferrovum* (37%). After the addition of lime milk, the water in the channel (W6) remained rich in *Gallionella* (40%) and *Ferrovum* (20%), but with *Porphyrobacter* (10%) newly emerging (Fig. 3A). The neutralization process continued in the sedimentation tank, where sample water W7 had a microbial community dominated by the unspecified methylotrophic bacterium *Methylobacterium-Methylorubrum* sp. (80%) growing mainly on organic carbon (42) (Fig. 3A). Interestingly, both *Methylobacterium* and *Porphyrobacter* have previously been found in biofilms on deteriorating historical paintings (43). Neutralization was associated with a significant reddish-brown mineral precipitation, indicative of polymetallic mineral formation with iron dominating and iron-oxidizing genera disappearing. Notably, *Methylobacterium* has previously been identified in biofilms on brown-colored coatings (43), suggesting its capability to thrive in alkaline environments enriched with iron.

Finally, water emerging from sediments (W8; Fig. S1), hosted *Gallionella* (10%) and heterotrophic bacterial representatives of the classes *Parcubacteria* (7%) and *Candidatus Omnitrophus* (7%; Fig. 3A). While uncultivable, *Candidatus Omnitrophus* metagenomic data describe it surprisingly well as an *Archea* capable of methane oxidation, hydrogen utilization, and/or dissimilatory nitrate reduction (44).

Overall, the microbial structure of waters from the Zlaté Hory mine, 30 years after closure, can be described as having low diversity but stable community composition, when compared with previously published data (13). The presence of FeOB in acidic mine waters presupposes active pyrite-ore weathering that could be due to an increase in FeOB metabolism, possibly leading to a gradual equilibrium as residual sulfidic ore is oxidized.

Sinter and sludge samples were collected from the same underground locations as their respective water samples (see Fig. S2 at https://doi.org/10.6084/m9.figshare.29616554), based on the visually promising formation of biofilms on the shaft walls. Other solid samples (i.e., sludge and sediment from the tailing site) were collected to assess changes in microbial community composition in response to (i) AMD treatment and (ii) increased toxic load in AMD processing end products. Across all solid samples, 20 bacterial genera demonstrated an abundance >1%.

The copper-rich sinter S1.1 (46 mg·kg^−1^) exhibited a relatively low bacterial abundance, being predominantly colonized by the as yet-uncultivated genus *KD4-96* (4%) and *Nitrospira* (13%; Fig. 3B). The most abundant genus was *Nitrospira* (Fig. 3B), which is primarily described as chemolithoautotrophic nitrite- and/or ammonium-oxidizing bacterium (comammox). Notably, copper resistance genes have previously been identified in comammox *Nitrospira*, but are absent in strictly nitrite-oxidizing *Nitrospira* strains (45), suggesting the potential presence of ammonia in the copper-rich sinter. In addition to nitrite and ammonia, *Nitrospira* is capable of utilizing alternative substrates such as molecular hydrogen and formate, using either oxygen or nitrate as a terminal electron acceptor (46, 47). This genus is also characterized by a high iron requirement, which it meets through siderophore-mediated uptake (47). Furthermore, *Nitrospira* has been implicated in steel corrosion, indicating its potential to extract electrons directly from metal surfaces (48). Though *Nitrospira* is not typically dominant in the microbial communities of former copper mines, it has been shown to become a major component in copper-contaminated grassland soils with increasing copper concentrations (49).

The presence of genus *KD4-96* (phylum *Chloroflexi*) has previously been associated with metal-contaminated soils and rhizospheric microbial communities tolerant to cadmium and arsenic (50, 51), and it has shown a strong correlation with aluminum and iron content in soft coal slags (52). The concentrations of iron (63 mg·kg^−1^), aluminum (154 mg·kg^−1^), and arsenic (49 mg·kg^−1^) detected in sinter S1.1 are consistent with previously reported associations.

Similarly, the zinc-rich sinter S1.2 (150 mg·kg^−1^), which also contained notable concentrations of iron (108 mg·kg^−1^) and copper (15 mg·kg^−1^), exhibited low overall bacterial relative abundance (Fig. 3B), which may be attributed to the known antibacterial properties of zinc (53, 54). The dominant taxon was the uncultivated genus *DSSD61* (11%), an ammonia-oxidizing member of the family *Nitrosomonadaceae*, accompanied by *Brevundimonas* (5%), *Nitrospira* (3%), and FeOB *Gallionella* (1%). Notably, *Brevundimonas diminuta* has been reported to overcome Zn(II) toxicity through immobilization via calcium carbonate (CaCO_3_) precipitation (55).

Sample S1.3, collected as pure copper, mainly comprised *Gallionella* (22%) and its close relatives (12%), *Rhodanobacter* (7%), and *KD4-96* (5%). The coexistence of FeOB and nitrate-reducing bacteria has previously been reported in autotrophic cultures (56). The proximity of iron-rich sediments (Fig. S2) suggests that these bacteria may have come from the sediment. There was a clear similarity in bacterial community composition between sample S1.3 and the iron-rich sludge at S2.1 (Fig. 3B). FeOBs, such as *Ferrovum* and *Gallionella*, which have previously been identified in metal-contaminated environments, mainly in stalks (57), were both detected in samples S2.1 and S2.2, appearing as a brownish sludge extruding from cracks in the tunnel walls, and as a brown-red mineral crust on these depositions (Fig. 3B).

Extremely acidophilic organisms, such as *Leptospirillum*, *Acidithiobacillus*, and *Acidocella* spp. (58, 59), usually present in AMD, were not observed in underground waters at the Zlaté Hory mine. However, *Acidithiobacillus, Acidiphilium, Acidimicrobia,* and acid-tolerant WPS-2 have all been detected in iron-containing stalks (S2.3) or crust (S2.4) together with the FeOB *Ferrovum* and *Metallibacterium*, the latter described as an acid-tolerant ammonium-producing facultative anaerobic bacteria (Fig. 3B). As such, it could neutralize the acidic environment for neutrophilic genera such as *Rhodanobacter*.

The iron-rich sludge obtained from the sedimentation tank exhibited a noticeably different microbial community, probably due to neutralization of AMD and close interaction with the surrounding environment (Fig. 3B). Dominant genera in this sample included *Hydrogenophaga* (22%), *Silanimonas* (17%), *Porphyrobacter* (14%), *Gallionella* (10%), and *Ralstonia* (10%). The bacterial community of this alkaline sludge is in line with previous findings, with a high abundance of the genus *Hydrogenophaga* having been found in hyperalkaline steel-slag-fill emulates and in hyperalkaline springs in Cyprus (60, 61). Interestingly, there was a relatively high abundance of the acidophilic genus *Gallionella*, which generally tolerate a lower pH (4-6) and microaerophilic conditions (62). Uninhibited growth of *Gallionella* has also been reported under neutral pH and fully aerated conditions, probably enabled due to inhibition of the autocatalytic iron oxidation caused by decreased chemical iron oxidation and low water temperature (63).

By contrast, sludge samples from the tailing site (S8.1 and S8.2), and the reddish sediment (S8.3) collected from beneath a leakage exhibited distinct microbial compositions (Fig. 3B). The dry sludge, originating from metal separation processes and as water treatment, showed a notable abundance of *Sphingomonas* (56%), *Rhodococcus* (34%), and *Thiobacillus* (29%), demonstrating their ability to overcome the toxic effects of cobalt, copper, and zinc (64–66). However, the DNA yield from sample S8.3 was significantly lower than the other two samples, indicating a sparse presence of heterotrophic microorganisms, representing <5% of the community (Fig. 3B).

Based on the results obtained, the relatively stable geochemical conditions observed in waters and sinters from the underground mining site at Zlaté Hory suggest that microbial community structure does not vary greatly over time. This stability is supported by comparisons with previous findings, which indicate that microbial communities tend to be representative of a site’s geochemistry (13). By contrast, microbial abundance may be influenced by seasonal changes at aboveground sites at the treatment plant and tailing site (67).

### Fungal biodiversity and community composition

Fungal community size was assessed through amplification of the ITS2 region and DNA sequencing, resulting in a total of 32,261 and 33,885 fungal ASVs identified in water and solid samples, respectively. Unfortunately, previous studies focusing on microbial colonization of underground mines in Zlaté Hory have not included data on fungal diversity (13, 15), thereby preventing comparisons.

**Fig 4:**
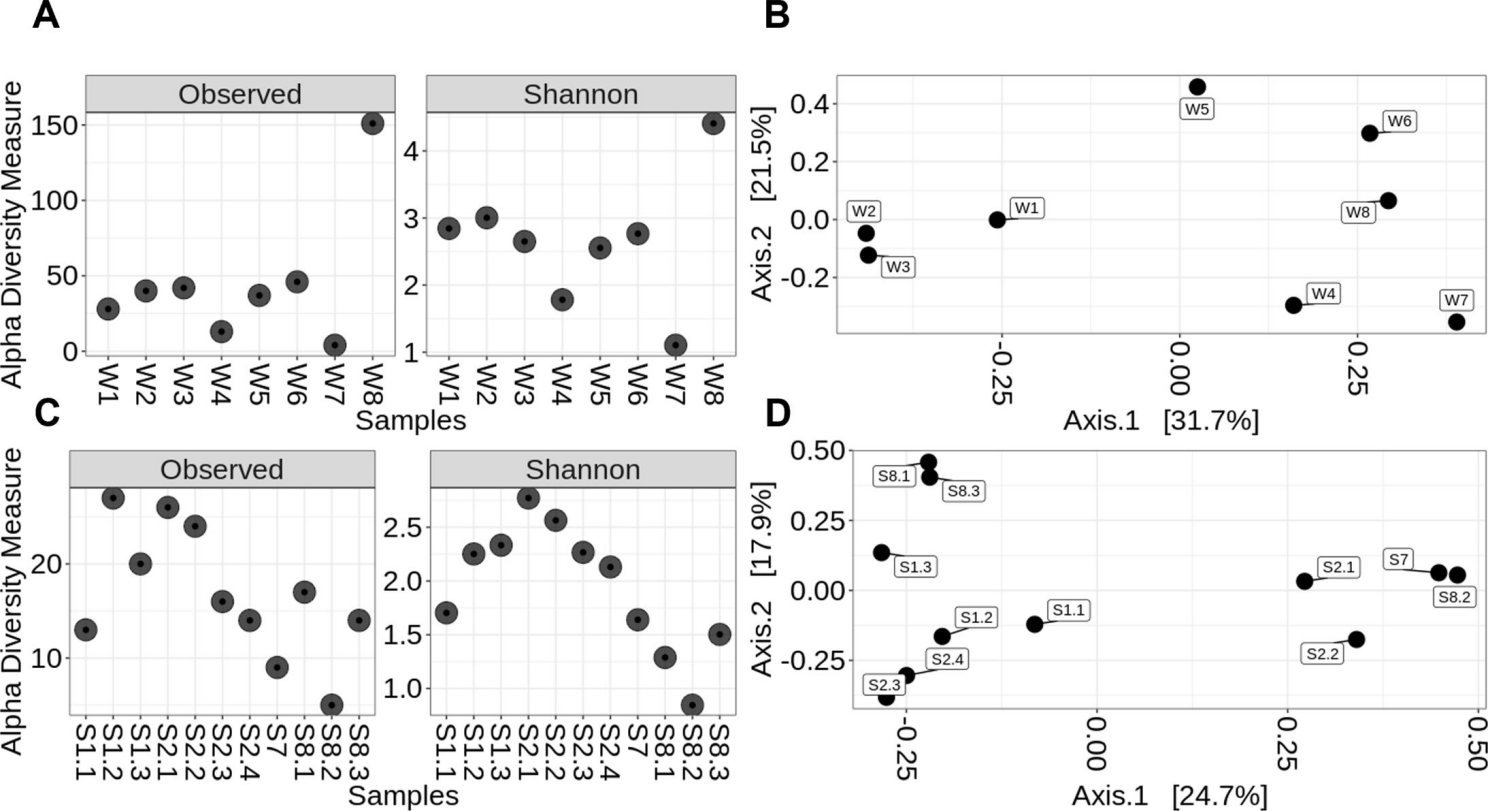
Alpha diversity (**A and C**) and PCoA (**B and D**) of bacterial communities in water (**A and B**) and solid (**C and D**) samples based on data from sequencing analysis using ITS rRNA primer targeting fungi.

Alpha diversity analysis of fungi in samples before and after treatment revealed generally low fungal diversity and richness, as measured by the Chao, Shannon, and Simpson indices. Nearly all water samples (W1 – W7) exhibited low diversity, with samples W4 and W7 having the lowest Shannon index observed. Interestingly, fungal abundance in sample W8 was significantly higher, with an index value exceeding 60 (Fig. 4A).

Beta diversity analysis at the genus level revealed a scattered pattern with a cluster comprising acidic water samples (W1, W2) and sample W3, and another cluster including water samples collected outside the mine together with sample W4 (Fig. 4B).

Fungal diversity in solid samples showed a decreasing trend moving towards the outside of the mine, with the lowest diversity detected for sample S8.2, this correlating with low-yield DNA content. A significantly higher Shannon diversity was observed for fungal community composition of metal-rich sinters (S1.2, S1.3, S2.1, S2.2, S2.3, and S2.4; Fig. 4C), suggesting a rich fungal community inside the mine. The highest Shannon diversity index was recorded in the iron-rich sample 2.1 (Fig. 4C).

Beta diversity analysis of solid samples based on genus-level similarities formed two distinct clusters (Fig. 4D), the first including copper- and zinc-rich sinter samples from acidic sites (S1.1, S1.2, S1.3, S2.3, and S2.4) and sludge samples S8.1 and S8.3, while the second comprised iron-rich samples from inside the mine (S2.1 and S2.2) and sludge sample S7, collected from the sedimentation tank (Fig. 4D).

Numerous studies have reported that fungi can be more tolerant to metals than bacteria, including actinomycetes (e.g., references [68, 69]). When comparing reference and copper/nickel-contaminated sites in Northern Ontario, Canada, Narendrula-Kotha and Nkongolo (39) found that the relative abundance of fungi, especially *Ascomycota*, became elevated at the contaminated site, whereas most bacteria, including actinomycetes, were suppressed by the presence of metals. Moreover, the same authors detected 44 fungal species at the contaminated site, among which 11 were specific to the site (39). However, at the Zlaté Hory mining site, we detected 22 genera with an abundance >1% in all water and solid samples, with 2-8 genera found in specific samples (Fig. 4). Fungal community composition differed significantly in water and solid samples, depending on chemical composition. For example, fungal abundance in acidic waters was relatively low (60%–70%), with most belonging to the phyla *Ascomycota, Basidiomycota,* and family *Mortierellaceae*. Some fungi, such as those of family *Mortierellaceae*, are normally incapable of overcoming heavy metal toxicity but have been shown to gain tolerance with the help of endosymbiotic bacteria (70).

**Fig 5:**
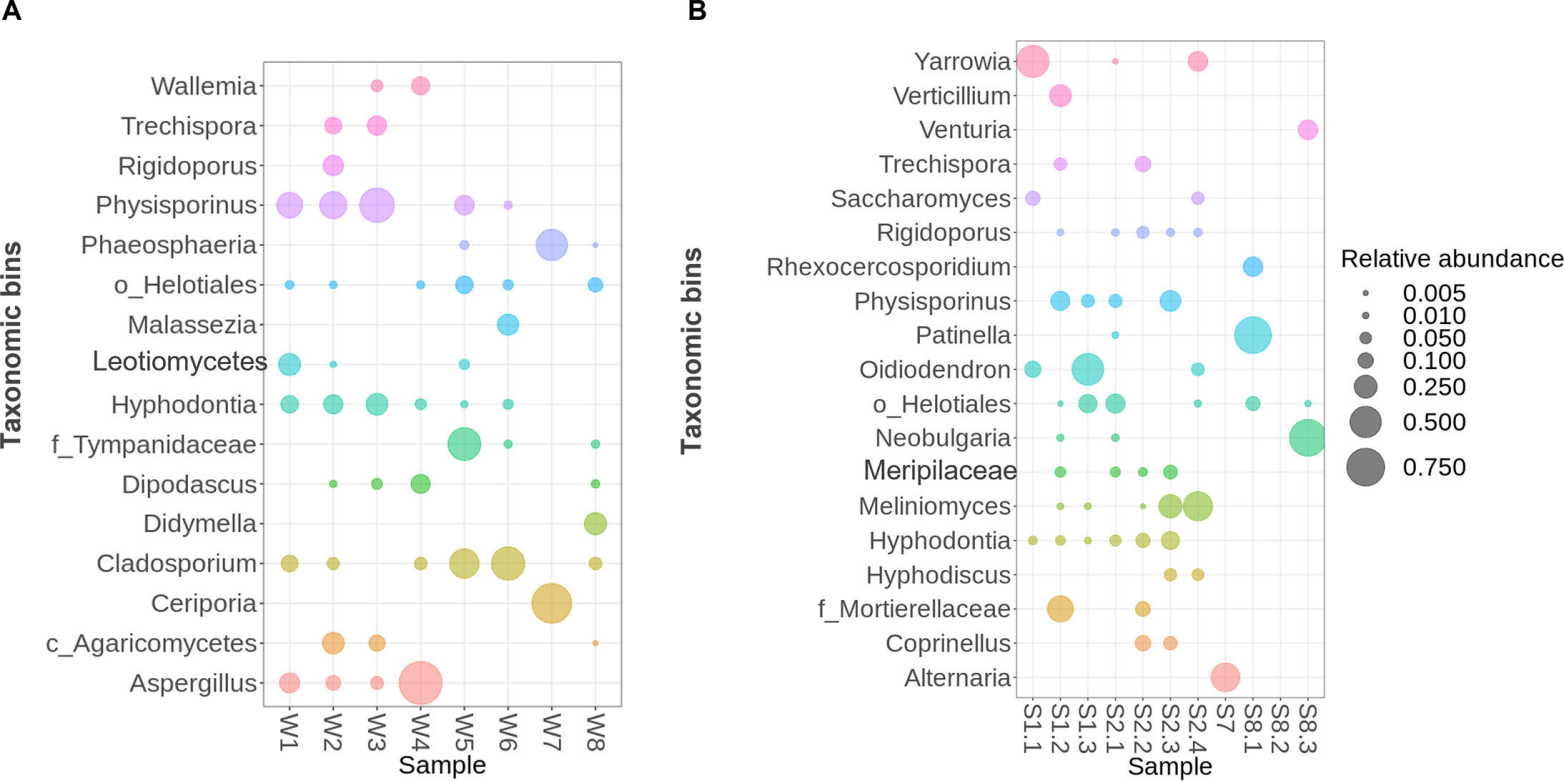
Relative abundance of fungal genera as detected by sequencing of the ITS rRNA in the water (**A**) and solid samples (**B**).

The composition of fungal genera in groundwater and mine-related samples was strongly influenced by pH and metal content, with acidic groundwaters (W1, W2, and W3) and the zinc-rich sinter sample (S1.2) exhibiting a high relative abundance of the genus *Physisporinus* (16%–35%; Fig. 5A). This genus is known for its ability to degrade lignin-rich substrates and has previously been found on the walls of a former gold mine in an arsenic-rich environment (71). In addition, the acidic sample W1 hosted the genera *Cladosporium* (6%) and *Hyphodontia* (7%; Fig. 5A), both of which include species previously reported in extreme or contaminated environments, suggesting a level of acid and/or metal tolerance (72).

By contrast, sample W2 from the ZH-West site, which has distinct geochemical conditions, with localized iron-rich contamination and circumneutral pH, hosted *Rigidoporus* in relatively high abundance of 6%–34% (Fig. 5A). This genus includes wood-decaying fungi important in organic matter turnover under stressed conditions. Genera such as *Physisporinus* and *Rigidoporus* were most likely introduced into the mine via the wooden supports used in the shafts and tunnels. A noticeably different fungal profile was observed in neutral pH groundwater at location W4, where *Aspergillus* (56%) and *Dipodascus* (9%) dominated (Fig. 5A). The high prevalence of *Aspergillus* is expectable, as several species within this genus are known for their resistance to heavy metals and their capacity to adapt to harsh environments, including mining sites (73). This suggests that *Aspergillus* may serve as a key fungal taxon in neutral but still metal-impacted waters.

While mine surfaces were colonized by a few fungal genera, they occurred at significant abundance, suggesting a high tolerance to toxic loads. Sample S1.1, for example, hosted three fungal species: *Yarrowia* (53%), *Oidiodendron* (11%), and *Saccharomyces* (9%). *Yarrowia lipolytica* has previously been reported to efflux copper (2 mM) due to increased acid phosphatase activity (74). Similarly, samples S1.2 hosted *Physisporinus* (17%), *Verticillium* (23%), and a representative of the family *Mortierellaceae* (33%; Fig. 5B). The high abundance of the latter taxon correlates with high abundances previously reported at other contaminated sites (75). The most abundant fungus in sample S1.3 was *Oidiodendron* (52%), which exhibits tolerance to copper and zinc (76, 77), and members of the order *Helotiales*, which have previously been found at metal-contaminated sites (78). The fungal community composition of samples S2.1 and S2.2 was similar to that of water sample W2, comprising fungi of the genera *Rigidoporus, Physissporinus,* and *Trechispora*. Both samples S2.1 and S2.2 exhibited around 45% unspecified fungal genera (Fig. 5B). Iron-rich stalks and crusts also contained *Meliniomyces* sp., which is recognized as a root-associating fungus, similar to *Oidiodendron*, and has previously been shown to withstand heavy metal toxicity, including uranium (79, 80).

Solid samples from the surface treatment-plant area exhibited a lower fungal community diversity, with sample S7, for example, dominated by the genus *Alternaria* (42%) along with a similar proportion of yet unspecified fungal genera. Sludge samples from tailings differed significantly from each other and from other samples, with sample S8.1 dominated by the genus *Patinella* (72%). Finally, a sample of iron-rich sediment (S8.3) showed a high abundance of *Neobulgaria* (71%) and *Venturia* (18%; Fig. 5B). *Neobulgaria* has previously been described as an arsenic-resistant fungus, having been found in decommissioned gold and thallium mines (81, 82).

### Metal tolerance of isolated bacteria and fungi

Heterotrophic microorganisms are well adapted to environments with high toxic loads and possess the capacity to process heavy metals through various mechanisms, including redox transformations, biosorption, and bioaccumulation (83–86). In this study, almost 90 bacterial strains and 20 fungal species were isolated from underground and surface solid samples (with varying amounts of organic matter) from the Zlaté Hory mine. Metal tolerance was evaluated by determining the MBIC of heterotrophic microorganisms (bacteria and fungi) isolated from iron-, copper-, and zinc-rich water (W1), sinter deposits (S1.1, S1.2, S2.1, and S2.2), and iron-rich sediment (S8.3). Thirty bacterial and 10 fungal isolates were then chosen for further analysis, based on their ability to tolerate heavy metals (Table 2).

**TABLE 2:** Minimum biofilm inhibitory concentration (MBIC) for zinc (Zn), copper (Cu), cobalt (Co), lead (Pb), and selenium (Se) in selected cultures isolated from underground and surface areas at the former mining site at Zlaté Hory

#	Microorganism	Identity (%)	Media	Source	MBIC (mM)						
					Zn	Cu	Co	Mn	Pb	As	Se
Bacteria from mine											
1	*Microbacterium phyllosphaerae* ^*e*^	99.23	NA^*a*^	W1_1b	4	2	2	n.d.^*d*^	2	n.d.	n.d.
2	*Uncultured Sphingopyxis* sp.^*e*^	100	NA	S1.1_2b	1	1	2	n.d.	8	n.d.	16
3	*Sphingobium* sp. 9 c7^*e*^	100	R2A^*b*^	S1.2_3b	4	8	n.d.	n.d.	8	n.d.	>8
4	*Variovorax* sp. SIB_Cd_R3^*e*^	99.67	R2A	S1.2_4b	8	>4	n.d.	n.d.	8	n.d.	>8
5	*Pseudomonas* sp. HA71^*e*^	99.71	NA	S2.1_5b	2	4	2	n.d.	8	n.d.	>8
6	*Bacillus pumilus* ^*e*^	100	NA	S2.1_6b	16	1	2	n.d.	8	n.d.	>16
7	*Sphingomonas alpina* ^*e*^	99.45	NA	S2.1_7b	8	4	4	n.d.	8	n.d.	>16
8	*Pedobacter* sp.^*f*^	–^*g*^	NA	S2.1_8b	>16	4	2	n.d.	8	n.d.	>8
9	*Microbacterium profundi* ^*e*^	99.76	NA	S2.1_9b	2	4	2	n.d.	8	n.d.	>16
10	*Bacillus licheniformis* ^*e*^	99.87	R2A	S2.2_10b	8	2	2	n.d.	8	n.d.	16
11	*Pseudomonas* sp.^*f*^	–	NA	S2.2_11b	1	4	4	n.d.	8	n.d.	16
12	*Variovorax boronicumulans* ^*e*^	99.91	R2A	S2.2_12b	8	4	4	n.d.	8	n.d.	16
13	*Variovirax boronicumulans* ^*e*^	100	R2A	S2.2_13b	16	4	4	n.d.	8	n.d.	8
14	*Microbaterium* sp. HaHa633^*e*^	99.87	NA	S2.2_14b	8	>4	>4	n.d.	>8	n.d.	>8
15	*Microbacterium profundi* ^*e*^	99.60	NA	S2.2_15b	8	4	2	n.d.	8	n.d.	16
16	*Virgibacillus* GJ_(1)^*e*^	99.75	NA	S2.2_16b	1	4	n.d.	n.d.	4	n.d.	>8
17	*Polaromonas aquatica* pol1^*e*^	99.84	NA	S2.2_17b	1	1	1	n.d.	1	n.d.	1
18	*Pedobacter panaciterrae* SCZ9^*e*^	99.75	NA	S2.2_18b	2	4	2	n.d.	2	n.d.	>16
19	*Arthrobacter pascens* L10-1^*e*^	95.67	R2A	S2.3_19b	4	4	4	n.d.	8	n.d.	1
20	*Arthrobacter* sp. MB118^*e*^	98.95	NA	S2.3_20b	8	4	2	n.d.	8	n.d.	>8
Bacteria from sediment											
21	*Pseudomonas mandelii* ^*e*^	100	NA	S8.3_21b	8	>4	>4	n.d.	8	n.d.	16
22	*Bacillus subtilis* ^*e*^	99.62	R2A	S8.3_22b	4	>4	>4	n.d.	8	n.d.	>4
23	*Janthinobacterium* sp. BWHT3^*e*^	99.54	NA	S8.3_23b	1	2	4	n.d.	8	n.d.	16
24	*Yersinia* sp.^*f*^	–	NA	S8.3_24b	1	2	2	n.d.	>8	n.d.	16
25	*Rahnella* sp.^*e*^	99.72	NA	S8.3_25b	1	1	2	n.d.	8	n.d.	16
26	*Uncultured Pseudomonas* sp.^*e*^	99.71	NA	S8.3_26b	1	4	4	n.d.	>4	n.d.	>8
27	*Uncultured Pseudomonas* sp.^*e*^	100	NA	S8.3_27b	1	4	4	n.d.	>4	n.d.	>8
28	*Uncultured Pseudomonas* sp.^*e*^	99.63	NA	S8.3_28b	1	2	4	n.d.	8	n.d.	>16
29	*Janthinobacterium svalbardensis* KCOM, J. tructae^*e*^	–	NA	S8.3_29b	1	4	4	n.d.	8	n.d.	4
30	*Pseudomonas* sp.^*f*^	100	NA	S8.3_30b	1	1	4	n.d.	8	n.d.	8
Fungi from mine											
31	*Penicillium* sp.^*f*^	99.97	PDA^*c*^	S1.1_1f	>4	>4	>4	>4	>4	>4	0.01
32	*Cladosporium* sp.^*f*^	100	PDA	S1.2_2f	>4	>4	>4	>4	>4	>4	>4
33	*Bjerkandera minispora* ^*f*^	100	PDA	S1.3_3f	>8	>2	>4	>8	>4	>16	>4
34	*Bjerkandera minispora* ^*f*^	95.51	PDA	S2.1_4f	>4	>4	>4	>4	>4	4	1
35	*Alternaria alstroemeriae* ^*f*^	100	PDA	S2.1_5f	4	2	2	>4	8	2	>16
36	*Bjerkandera adusta* ^*f*^	100	PDA	S2.1_6f	>4	>1	>4	>4	>4	>4	>4
37	*Mucor hiemalis* ^*f*^	100	PDA	S2.3_7f	>8	>4	>4	>8	2	8	1
Fungi from sediment											
38	*Alternaria alstroemeriae* ^*f*^	100	PDA	S8.3_8f	>4	1	4	>4	4	2	1
39	*Trichoderma* sp.^*f*^	84.13	PDA	S8.3_9f	>8	4	8	>8	4	2	16
40	*Fusarium* sp.^*f*^	99.59	PDA	S8.3_10f	>8	2	1	>8	4	4	1

^*a*^ NA, nutrient agar.

^*b*^ R2A, R2A agar.

^*c*^ PDA, potato dexrose agar.

^*d*^ n.d., not determined.

^*e*^ Sanger sequencing.

^*f*^ NGS, next-generation sequencing.

^*g*^ –, not applicable.

MBIC analysis revealed that many of the bacterial isolates exhibited tolerance to several metals, consistent with observations in previous studies (87–90), with lead and selenium identified as most tolerated among all isolates. By contrast, tolerance to zinc, copper, and cobalt varied depending on sampling habitat, likely reflecting species-specific or even strain-specific adaptations. For example, two distinct strains of *Variovorax boronicumulans* isolated from the same iron-rich wall sinter showed different MBICs for zinc and selenium (Table 2). Similarly, several isolates of as yet-uncultured *Pseudomonas* spp. from iron-rich sediment were distinguished as different strains based on distinct MBIC profiles (Table 2; cultures #26, #27, #28, and #30). Conversely, *Polaromonas aquatica* (#17) exhibited overall low tolerance for heavy metals and selenium (MBIC 1 mM for all metals tested).

In microorganisms, selenium tolerance, particularly resistance to selenite and selenate, is often associated with broader heavy metal tolerance, likely due to overlapping detoxification pathways and stress response mechanisms (12, 91–93). Most of the bacterial isolates tested grew successfully in the presence of selenium concentrations of >8–16 mM (Table 2). Indeed, many bacteria, belonging to the genera *Pseudomonas, Pedobacter, Microbacterium, Arthrobacter*, and *Bacillus,* are known for their ability to reduce selenate [Se(VI)] and selenite [Se(IV)] to elemental selenium [Se(0)], resulting in the formation of characteristic red precipitates (94, 95).

The highest MBIC value for lead was observed for culture #14 *Microbaterium* sp. (>8 mM), isolated from an iron-rich wall sinter, most other isolates only tolerating lead up to 8 mM (Table 2). These findings align with previous reports of strong lead tolerance in microorganisms isolated from heavy metal-contaminated soils, including *Microbacterium resistens*, *Bacillus pumilus*, and *Pseudomonas* sp. I3 (96, 97). Comparable results were also reported by Choinska-Pulit et al. (98), who found that *Pseudomonas azotoformans*, isolated from a contaminated site, exhibited a minimum inhibitory concentration (MIC) for lead of 1,000 mg·L⁻¹. Interestingly, our *Pseudomonas* isolate from the Zlaté Hory mine displayed an even higher lead tolerance.

Tolerance to zinc, copper, and cobalt varied markedly among the cultivated strains. Highest MBIC (>16 mM) for zinc recorded for *Pedobacter* sp., isolated from iron-rich sludge (S2.1), despite this only containing 2.6 mg.kg^−1^ zinc. Interestingly, high tolerance for zinc (>8 mM) was observed in three bacterial isolates from zinc/iron-rich sinters (S1.2 and S2.2), that is, #3 (*Sphingobium* sp.), #4 (*Variovorax* sp.)*,* and #14 (*Microbacterium* sp.), suggesting environmentally driven adaptation (Table 2).

**Fig 6:**
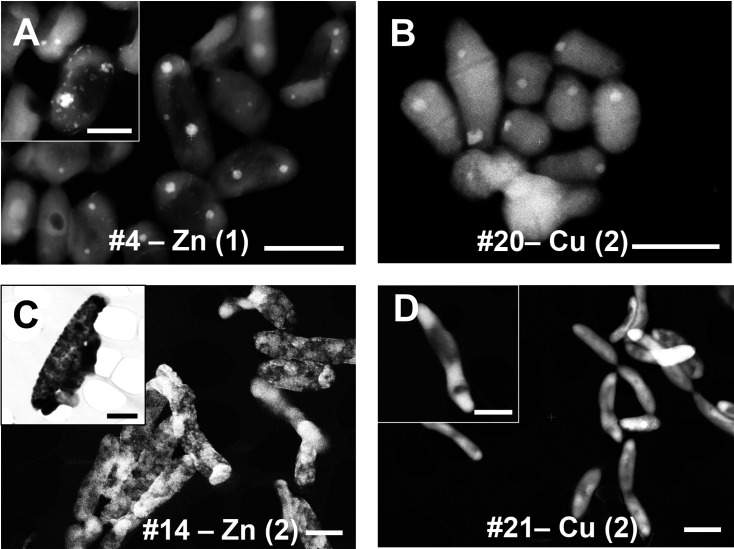
Detection of metal localization, determined by STEM-HAADF and growth curves for selected strains and metals. (**A**) Micrograph of culture #4 incubated with 1 mM Zn(NO_3_)_2_ (scale bar = 1 µm), *inset* = zinc nanoparticles (scale bar = 500 nm). (**B**) Micrograph of culture #20 incubated with 2 mM Cu(NO_3_)_2_ (scale bar = 1 µm). (**C**) Micrograph of culture #14 incubated with 2 mM Zn(NO_3_)_2_ (scale bar = 1 µm). (**D**) Micrographs of culture #21 incubated with 2 mM Cu(NO_3_)_2_ (scale bar = 1 µm).

Some of these highly tolerant isolates were tested further using HAADF-STEM (High-Angle Annular Dark-Field Scanning Transmission Electron Microscopy) to verify their ability to form metal nanoparticles and process metals intracellularly. In culture #4 (*Variovorax* sp.), we observed production of zinc particles localized within the cell in a few circular bodies. Zinc nanoparticles were eventually excreted and adsorbed onto the cell surface (Fig. 6A, inset). As previously reported, *V. boronicumulans* exhibits strong bioremediation potential by removing lead, cadmium, and zinc (86). Similarly, *V. paradoxus* from mine spoil has demonstrated high tolerance to zinc, copper, cadmium, and silver (88).

Discrete intracellular localization of copper within circular bodies was also observed in *Arthrobacter* sp. (Fig. 6B), with subsequent energy dispersive spectroscopy (EDS) confirming copper accumulation in these structures (see Table S4; Fig. S4 at https://doi.org/10.6084/m9.figshare.29616554). By contrast, culture #14 (*Microbaterium* sp.) revealed zinc nanoparticle accumulation and adsorption distributed across the entire cell surface (Fig. 6C), with EDS again verifying zinc presence within and on cells (Table S4; Fig. S4). The ability of this genus to accumulate and/or process zinc has been reported previously (12, 85).

By contrast, culture #21, *P. mandelii* isolated from iron-rich sediment (S8.3) showed no localized intracellular copper accumulation (Fig. 6D, inset; Table S4; Fig. S4), suggesting a lack of redox-active systems, despite the strain’s apparent capacity to withstand toxic environmental conditions through alternative mechanisms.

All fungal isolates exhibited high tolerance to zinc and manganese. *Alternaria alstroemeriae* was found in both above- and underground environments but showed variable responses to the metals tested. While *Bjerkandera minispora* and *B. adusta* are closely related species, *B. minispora* demonstrated greater metal tolerance than *B. adusta*. Among the isolates, *A. alstroemeriae*, *Mucor hiemalis*, *B. minispora*, and *Trichoderma* sp. all exhibited notably higher tolerance to metals compared to other fungi. Interestingly, *Penicillium* sp. did not show tolerance to selenium, while two fungal strains isolated from iron-rich sediment (S8.3) with a zinc concentration of 880 mg.kg^−1^, were found growing on a substrate with 8 mM zinc.

Though specific studies on *B. minispora* and *B. adusta* are limited, other members of the *Bjerkandera* genus are known for their ability to degrade pollutants and tolerate certain heavy metals, suggesting their potential use in bioremediation. Similarly, while direct studies on *A. alstroemeriae* are scarce, other species within the *Alternaria* genus, such as *A. chlamydosporigena*, have demonstrated tolerance to cadmium, lead, zinc, and copper (99). *Mucor hiemalis* has also shown strong potential in heavy metal biosorption, having been shown to effectively remove metals such as cobalt, chromium, copper, nickel, lead, and zinc from contaminated environments (100, 101). Species within the *Trichoderma* genus are also well documented for their metal tolerance, with some strains capable of removing up to 68% of nickel from culture media (102). *Trichoderma koningii* Tk10 has also been shown to have high tolerance to copper (up to 5.4 mM) (103), while *T. harzianum* can tolerate cadmium concentrations up to 300 ppm (104), and *T. asperellum* strain ZZY effectively reduces Pb²^+^ levels through competitive adsorption (105). Though *Penicillium* sp. Showed poor tolerance for selenium, other species, such as *P. simplicissimum*, have shown strong biosorption capacities for zinc and copper, indicating strain-specific variability (99).

Bacterial and fungal tolerance to heavy metals reveals significant differences in their abilities to withstand and adapt to metal stress, largely due to larger surface areas providing higher metal sorption capacities (106). Differences in metal tolerance, even among closely related bacterial strains, suggest that resistance is not solely species dependent but may also be significantly influenced by environmental and epigenetic factors, with local environmental pressures shaping the expression of metal resistance traits in microbial communities. Together with bacteria, fungi are able to form highly structured biofilms thanks to their filamentous structure, with extracellular polymeric substances and a unique microenvironment likely helping to sustain toxic loads (107, 108).

### Conclusion

This study provides a comprehensive geochemical and microbiological analysis of a former mining site in Zlaté Hory, Czech Republic, highlighting the relationship between geochemical conditions and microbial communities in mine-impacted environments. Geochemical characterization of mine waters, encompassing parameters such as pH, metal concentrations, and mineral composition, is crucial for understanding the formation and environmental impacts of AMD. The AMD treatment process resulted in the precipitation of iron and other metals, which co-precipitated with varying efficiency, leading to the accumulation of heavy metals and iron in solid waste. MBIC screening of isolated bacterial and fungal monocultures demonstrated the remarkable ability of microbiota to withstand and potentially transform heavy metals. Based on the microbial taxa identified and their known metabolic capabilities, we suggest that several water treatment processes at the site could be mediated by these bacteria.

First, Fe(II)-oxidizing bacteria (FeOB) present in the mine water could help reduce the toxicity of Fe(II) dissolved from pyrite. However, this oxidation process generates reactive oxygen species (ROS) (109, 110), which may be detrimental to other microorganisms, potentially explaining the observed low microbial diversity in these waters. FeOB were particularly abundant in the water samples and iron-rich wall sinters originating from polymetallic sections of the mine (W1, W2, S2.1, and S2.2), whereas samples from the former gold/copper mine (W3 and W4) lack detectable FeOB populations (Fig. 3).

Dissolved Fe(III) in mine water can lead to several geochemical and biogeochemical processes. As a strong oxidant, Fe(III) can further oxidize pyrite abiotically, initiating a feedback loop that sustains acid generation and promotes the mobilization of metals from host rocks. In addition, hydrolysis of Fe(III) in water releases hydrogen ions, resulting in the formation of iron hydroxide (Fe(OH)_2_) precipitates. This reaction contributes significantly to lowered pH conditions and exacerbates the solubility and toxicity of metals in mine waters. During the Zlaté Hory mine-water treatment process, the addition of lime milk induced precipitation of Fe(III) as iron hydroxide, facilitating co-precipitation of other metals and metalloids (e.g., arsenic) through adsorption. This process may be further enhanced by the presence of heterotrophic, metal-tolerant bacteria capable of adsorbing metal complexes onto their cell surfaces, particularly in water samples with higher pH. Bioremediation efforts could be supported by the activity of iron-reducing bacteria (IRB), which help neutralize acidity by reducing Fe(III) into Fe(II), thereby promoting further metal precipitation. Unfortunately, IRB was not detected in the groundwaters or deeper layers of sediment or sludge at the site.

Second, high sulfate concentrations suggest the presence of sulfate-reducing bacteria (SRB), which may contribute to the precipitation of metal sulfides, thereby reducing heavy metal mobility, particularly in anoxic or microaerophilic tailing zones (S8.1, S8.2, and S8.3). This hypothesis is supported by the low concentrations of toxic metals observed in the stream water (W8). The presence of the sulfur-oxidizing bacteria *Sulfurifustis* in sample W4 and *Thiobacillus* in samples S8.1 and S8.2 (Fig. 3) provides additional evidence of active sulfur cycling at the site and supports the occurrence of sulfate reduction.

Finally, heterotrophic bacteria capable of using organic matter of differing complexity (including xenobiotics) were abundant, particularly in samples with circumneutral to alkaline pH. Most have previously been shown to exhibit heavy metal tolerance or immobilization capability, for example, *Rhodococcus, Porphyrobacter*, *Methylobacterium*, and *Hydrogenophaga* in drainage samples from a Brazilian copper mine (111–114). Growth of heterotrophs is usually supported by metabolic by-products of nitrifying chemoautotrophs such as nitrite-oxidizing *Nitrospira* and the ammonia-oxidizing genus *DSSD61* (115, 116), found in wall sinters inside the mine (S1.1 and S1.2), accompanying the heterotrophic genera *KD4-96* and *Brevundimonas*, respectively, thereby closing the nitrogen cycle. This nitrogen cycle activity indirectly supports bioremediation processes by sustaining populations of heterotrophic bacteria, which are themselves capable of manipulating heavy metals. The detection of nitrifying and denitrifying bacteria (e.g., *Nitrospira, Silanimonas, Sphingomonas, DSSD61, Rhodanobacter*) in several samples could further indicate active nitrogen cycling. Though nitrate concentrations were below the detection limit of ICP, the presence of these bacteria suggests a potential contribution to overall water quality improvement.

Fungal diversity in both water and solid samples was similarly shaped primarily by geochemical factors such as pH and metal content. Acidic conditions were generally associated with reduced fungal diversity, whereas some neutral or metal-rich sites supported more abundant and specialized fungal populations.

To summarize, our data support the enormous potential of autochthonous microbial communities to tolerate and adapt to heavy metal pollution. Furthermore, our molecular-genetic data identified microorganisms involved in the cycling of basic earth elements, including sulfur, iron, and nitrogen. While active sulfur and nitrogen cycling, along with FeOB abundance, suggest natural biogeochemical processes, the absence of IRBs represents an imbalance in the iron cycle, limiting natural acid neutralization and metal precipitation under certain redox conditions. At the same time, chemical treatment (lime milk) proved effective at precipitating Fe(III) and other metals. Promotion of growth in metal-tolerant heterotrophs via nitrification products, along with their ability to adsorb metals at higher pH, indicates a synergy between the heterotrophic community, the nitrogen cycle, and engineering interventions. Overall, the balance is dynamic and will be influenced by both geochemical conditions and the presence/absence of key microbial groups, with some natural processes (e.g., SRB activity, heterotroph support by nitrifiers) contributing to remediation, while others (e.g., absence of IRB, ROS from iron redox conversions) complicate it.

This study provides an overview of isolated heterotrophic microorganisms and their ability to overcome metal(loid) toxicity. Interestingly, most of the isolated bacteria demonstrated an ability to tolerate metal concentrations higher than those found in the environment. The presence of metal-tolerant fungi in both underground and surface samples highlighted their ecological resilience and potential functional role in metal transformation and bioremediation. Moreover, microscopic analysis of the isolates revealed differences in heavy metal processing.

To implement environmentally friendly strategies and reduce the economic costs of cleaning up former mining sites, it would be more effective to optimize or reconfigure ongoing biogeochemical processes through engineering approaches (e.g., controlling redox conditions, supplying substrates, and potentially bioaugmenting with beneficial microorganisms) to enhance the efficiency of natural bioremediation mechanisms and offset those that hinder remediation in their natural state. Our findings provide a foundational understanding of fungal dynamics in a heavily contaminated mining environment and highlight the importance of integrating fungal community analysis into remediation planning and ecosystem assessments. Fungi can also support phytoremediation by improving soil structure, metal stabilization, and plant health in contaminated environments. Further investigation into microbiota in environments contaminated with multiple pollutants will be essential for increasing our understanding of microbial adaptation mechanisms and applying these findings to advanced remediation strategies.

## MATERIALS AND METHODS

### Description of the Zlaté Hory mining site

The former mining site at Zlaté Hory lies in the Jeseník district of Northern Moravia in the Czech Republic (50°12–13′N, 17°23–24′30″E). The ore district in Zlaté Hory consists of non-contrasting ore bodies of sulfide ores with a predominance of pyrite and accompanying arsenopyrite, chalcopyrite, sphalerite, galena, and gold. The mineralization of the interstitial character is mainly linked to the quartzite horizon, where the ore bodies show distinct zonation, with Cu-ores forming the central and deeper parts of the ore zones and Pb-Zn ores forming the edges of the bodies. The mineral composition of the rock bed in Zlaté Hory is primarily composed of pyrite-chalcopyrite or chalcopyrite-pyrrhotite sulfidic ores (117).

The Zlaté Hory mine currently consists of a system of horizontal, or nearly horizontal, tunnels excavated into a mountainside to provide direct access to the ore body, and vertical shafts or inclined excavations connecting the surface to deeper levels. The mine contains four nonferrous metal ore bodies (termed South, Hornické skály, East, and West), which were historically exploited for their rich gold and copper veins, with mining activities dating back to the 13th century (118, 119). Currently, polymetallic sulfide ores are dispersed in metamorphic Devonian volcano-sedimentary rocks. In addition to various metal sulfides, the ore contains oxide-based minerals such as rutile and anatase, as well as uraninite, minor phosphates such as xenotime, arsenic-bearing minerals such as cobaltine, and calcite along with extensively ionized carbonates (120).

The modern history of the region began in 1952 with the initiation of drilling exploration of the Zlaté Hory (ZH) deposits. These were subsequently divided into four parts: the first two parts comprising ZH-South and ZH-Hornické skály, known for their copper deposits; the third part, ZH-East, containing copper, lead, zinc, and silver deposits, while the fourth, ZH-West, containing deposits of copper, lead, zinc, and gold (119). The total mining area spanned 3.1 km², with deposits accessed via four pits, 14 adits, four decline shafts, and 11 chimneys. Modern mining operations commenced at ZH-South in 1965, at ZH-Hornické skály in 1981, at the ZH-East polymetallic deposit (containing sphalerite, chalcopyrite, pyrite, and galenite [119]) in 1988, and at ZH-West in 1990 (118).

Mining activities in the Zlaté Hory region have now been discontinued, with the extraction of monometallic copper deposits ceasing in 1990, and operations at the ZH-East deposit ending in 1992. The ZH-West polymetallic deposit, which focused primarily on gold with smaller quantities of copper and zinc, was mined between 1990 and 1993. Mining operations, which were based on open chamber mining, resulted in the extraction of 7,184.4 kt of ore (121). The extracted ore was processed locally using selective froth flotation to produce zinc or gold/copper concentrates (13).

During the operational period of the mine (until 1995), mine water was neutralized underground, and the resulting sludge was deposited in a tank in front of the dewatering gallery. This sludge was then pumped to a tailings pond together with flotation sands from the water treatment plant (119). Decommissioning activities were carried out between 1990 and 2005, beginning with the ZH-South and ZH-Hornické skály deposits, followed by the closure of the ZH-East polymetallic deposit and the ZH-West mine. In 1998, pumping of water from the deposit’s 5th level ceased, allowing water to gradually rise to the level of the drainage gallery (3rd level), from which it began to discharge from the mine by gravity (119).

### Mine water treatment

Approximately 20% of annual atmospheric precipitation (1,000 mm/year) contributes to AMD formation, which is generated from rainwater infiltrating through fissure systems, abandoned mine workings, and old underground boreholes and through groundwater discharge from fault and fracture systems. As this water passes through the deposit, it dissolves residual ore, thereby becoming enriched with metals. The resulting waters are strongly mineralized and acidic due to oxidation of pyrite, chalcopyrite, and arsenopyrite (equations 1–3) (119, 122). The entire AMD generation process can be described via the following chemical equations:


(1) $$FeS_{2}+H_{2}O+7/2O_{2}\to FeSO_{4}+H_{2}SO_{4}$$

(2) $$CuFeS_{2}+4O_{2}\to CuSO_{4}+FeSO_{4}$$

(3) $$2AsFeS+7O_{2}+H_{2}O\to 2FeAsO_{4}+2H_{2}SO_{4}$$

(4) $$2FeSO_{4}+4O_{2}\to Fe_{2}(SO_{4}{)}_{3}$$

(5) $$Fe_{2}(SO_{4}{)}_{3}+6H_{2}O\to 2Fe(OH{)}_{3}+3H_{2}SO_{4}$$

The dissociation of pyrite increases acidity due to the further oxidation of ferrous sulfate (FeSO4) into unstable ferric sulfate (Fe_2_(SO_4_)_3_; equation 4), which hydrolyzes into ferric hydroxide (Fe(OH)_3_) and sulfuric acid (H_2_SO_4_; equation 5). The rising concentration of ferric iron accelerates further sulfide oxidation and rock weathering (119). The AMD are classified into four classes according to acidity and concentrations of iron, aluminum, and sulfates (20).

An AMD treatment plant was constructed at the site between 1990 and 1998 (Fig. 1). AMD exits the mine through a single outlet shaft, and this is neutralized by the addition of lime milk (Ca(OH)_2_), raising pH from approximately 3.5–4.0 to 9.5. The mine water is periodically aerated to promote the precipitation of dissolved metals in the form of sludge, as shown in the reactions below (equations 5–8) (119). The resulting concentrated sediment is then collected in a drainage groove.


(6) $$H_{2}SO_{4}+Ca(OH{)}_{2}\to CaSO_{4}+2H_{2}O$$

(7) $$Fe_{2}(SO_{4}{)}_{3}+3Ca(OH{)}_{2}\to 2Fe(OH{)}_{3}+3CaSO_{4}$$

(8) $$CuSO_{4}+Ca(OH{)}_{2}\to Cu(OH{)}_{2}+CaSO_{4}$$

(9) $$ZnSO_{4}+Ca(OH{)}_{2}\to Zn(OH{)}_{2}+CaSO_{4}$$

Finally, the treated water is directed through a secondary sedimentation tank and discharged into a tailing pond. The sludge (annual production: 2,500 tons of wet sludge) is regularly removed and deposited on-site, while the treated water is released into the Zlatý Potok stream (118). Each year, approximately 3 million m³ of mine water undergo treatment (119).

### Sampling process

Samples of nonfiltered mine water, treated water, swabs from mine walls, sludge from the nearby tailing site, and sediment leaking from the tailing site were collected during two campaigns in the summer of 2023. To capture microbial diversity in relation to geochemical and mine water characteristics, samples were collected from different locations within the underground shafts. In total, four underground mine water samples were collected, all from horizon 3 (depth 200 m, 538 m a.s.l.), including water from a former copper/gold mine emerging from the ceiling of a shaft at the third level, referred to as the “Blue Shaft.” This water was mixed with inflows from the mine’s polymetallic section. The sampling locations (see Fig. 1) and their characteristics can be described as follows:

- W1 (ZH-South): acidic water sample from gold mining activities.
- W2 (ZH-West): acidic water sourced from a former gold mine.
- W3 (ZH-East, Pit No. 3): mixed water from former copper and gold mines.
- W4 (ZH-East): pure water dripping from a borehole in the ceiling of a tunnel forming a turquoise precipitate on the tunnel walls, indicative of former copper presence.

Four additional samples were collected at different stages of the water treatment process:

- W5 (Drainage Shaft Outflow): outflow of the drainage shaft containing mixed water from gold, copper, and polymetallic mines.
- W6 (Neutralization Station): alkaline water obtained after lime milk treatment.
- W7 (Sedimentation Tank): water with a brownish-yellow precipitate.
- W8 (Tailing Leakage): water emerging through sediment leakage below the tailing site.

In addition, sinters from the tunnel walls, sludge, and sediment were taken from respective locations (Fig. 1) and described as follows:

- S1.1: turquoise mineral (described as allophane [13]) precipitated on the wall near W1.
- S1.2: white mineral (zinc-rich) precipitated on the wall near W1.
- S1.3: pure copper precipitate, partially oxidized, found on the floor in a shaft alongside W1.
- S2.1 and S2.2: brown sediment deposited on the shaft wall (as at W2).
- S2.3: gelatinous iron-rich stalactites of brown-red color on the wall near the gold mine stream in ZH West.
- S2.4: crust on black-brown colored sludge.
- S7: orange to brown sediment from the sedimentation tank.
- S8.1 and S8.2: samples of dried sludge drilled from the tailing site originating from (i) the processing of ore at the mining site and (ii) from the treatment of mining water.
- S8.3: sediment leaking from the tailing site.

Water samples intended for microbiological analysis were collected in sterile 500 mL screw-capped bottles, with a total volume of 1–3 liters, transported to the laboratory under controlled conditions (4°C). Approximately 1 L of each water sample was filtered *in situ* using 0.22 µm Sterivex sterile filters (Merck, Germany). These filters were then stored at −20°C to preserve DNA integrity until extraction. Samples designated for cultivation were refrigerated and processed within 48–72 hours, then frozen immediately for genetic analysis. Sludge samples were collected in triplicate from an area of approximately 10 cm^2^ using sterile equipment and placed into sterile sample tubes. Sludge samples were processed similarly, that is, processed within 48 hours for cultivation then frozen immediately for genetic analysis. The dry mass weight of sludge was determined by drying the samples in an oven at 110°C for 2 hours. One gram of dry mass was used for cultivation, and 5 g was allocated for genetic analysis. The dried samples were also used for chemical analysis.

### Water chemistry

Physicochemical parameters of the water samples, including temperature, pH, redox potential (Eh), dissolved oxygen, and conductivity, were measured on-site using a WTW 3110 pH meter (WTW, Germany). pH and temperature were measured with a SenTix 980 combined IDS electrode, and redox potential with a SenTix ORP-T 900 Pt – Ag/AgCl IDS redox electrode. Dissolved oxygen was measured using a WTW FDO 925 IDS probe, and conductivity using a WTW TetraCon 925 IDS probe (WTW, Germany). Chemical composition of the water samples was determined by measuring the total content of metals and anions using inductively coupled plasma mass spectrometry (ICP-MS), targeting 19 anions and 7 cations after acidification with concentrated suprapure nitric acid.

### DNA extraction and 16S rRNA sequencing

DNA was extracted from filters using the DNeasy PowerWater kit (Qiagen, Germany), following the manufacturer’s protocols. DNA concentration was quantified using a Qubit 2.0 fluorometer (Thermo Fisher Scientific, USA) according to the manufacturer’s guidelines. The DNA samples were then stored at 4°C until further processing. The bacterial 16S rRNA gene variable region V4 was amplified using the primers 515F (5′-GTGCCAGCMGCNGCGG-3′) (123) and 802R (5′-TACNVGGGTATCTAATCC-3′) (124). For fungal community analysis, the Internal Transcribed Spacer (ITS) rRNA gene region was amplified with the primers ITS3F (5′-GCATCGATGAAGAACGCAGC-3′) and ITS4R (5′-TCCTCCGCTTATTGATATGC-3′) (125). Amplicon sequencing was performed on the Genexus Integrated Sequencer (Thermo Fisher Scientific, USA) using the Genexus sequencing combo kit, Ion GX5 chip, GXS coupler kits, and GXS template strips (3-GX5 and 4 combo). To refine the taxonomic resolution of isolated strains, Sanger sequencing was performed using the primers for bacterial 16S, forward primer 16S-6F (5′-AGAGTTTGATCMTGGCTCAG-3′) and reverse primer 16S-1234R (5′-CCCACCTTCCTCCGTTTTGTCAA-3′). In addition, the forward primer ITS1F (5′−TCCGTAGGTGAACCTGCGG−3′) and reverse primer ITS4R (5′−TCCTCCGCTTATTGATATGC−3′) were used for the fungal ITS gene (126).

### Data analysis

The data obtained were processed using QIIME 2 v. 2024.2 software (127). First, the raw sequence data were demultiplexed and quality-filtered using the q2-demux plugin, after which denoising was performed with DADA2 (via q2-dada2) (128). This method denoises single-end sequences, dereplicates them, and filters out chimeras. Taxonomy was assigned to amplicon sequence variants (ASV) using the q2‐feature‐classifier (129), and classified by classify-sklearn naive Bayes against the Silva 138 database (130), after which mitochondria and chloroplasts were removed from the data set. The accuracy of classification was evaluated against an artificial MOCK community sample.

QIIME 2 outputs were processed using the phyloseq R package (131). Alpha diversity metrics (Observed, Shannon, Simpson, and Inverse Simpson) were computed on rarefied (sub-sampled without replacement) data. Principal coordinates analysis (PCoA) was used to compare bacterial communities across samples, while Bray-Curtis distance metric was employed to measure differences between communities based on their relative abundances without rarefaction. In addition, taxonomy bubble plots were created using the same relative abundances but including taxonomic bins only (aggregated at genus level) with a mean relative abundance >0.01.

### Cultivation

One gram (dry weight) of the solid samples (S1.1, S1.2, S2.1, S2.2, and S8.3) was resuspended in 10 mL of phosphate-buffered saline (PBS; pH 7.0) and then incubated at 30°C for 2 hours while shaking at 135 rpm. Spread plates of nutrient agar (DSM 1 a), R2A agar (DSM 830), and Luria-Bertani agar (DSM 381) at 100% and 10% concentration, along with *Sphaerotilus-Leptothrix* medium (DSM 803) for the cultivation of FeOB, were prepared in triplicate by inoculating 0.1 mL of the samples or serial dilution (10⁻¹–10⁻^4^), spread using a sterile glass rod (132). All media were supplemented with the antifungal agent cycloheximide (40 µg mL^−1^). Plates were then incubated at 28°C for 3, 5, and 7 days. The purity of monocultures was verified using Gram staining and Live/Dead staining using the Live/Dead BacLight bacterial viability kit (Invitrogen, USA) and an AxioImager epifluorescence microscope (Zeiss, Germany). The isolated monocultures were preserved at −80°C in media supplemented with 60% (vol/vol) glycerol.

Fungal isolation was carried out using the serial dilution method as described for bacterial isolation, with inoculation of potato dextrose agar (PDA) plates supplemented with tetracycline (100 µg mL⁻¹) to inhibit bacterial growth, following the protocol described previously (133). Plates were sealed and incubated at room temperature for 5–7 days until fungal growth was observed. Fungal colonies were chosen for further purification according to variations in colony morphology, color, and surface texture.

### Determination of MBIC

Metal tolerance was assessed by determining the MBIC on agar plates supplemented with copper, cobalt, selenium, lead, and zinc salts (Table S5). Serial threefold dilutions of selected elements, ranging from 0 mM to 16 mM, were prepared and tested using bacterial suspensions adjusted to approximately 3 × 10⁸ CFU/mL (equivalent to 1 McFarland standard). Nutrient agar or R2A agar plates were selected according to the nutritional preferences of bacterial strains, as some were unable to grow on minimal salt media. All assays were performed in triplicate. MBIC values for fungal monocultures were also determined on solid media, using PDA plates supplemented with increasing concentrations of the respective metal or metalloid. MBIC was defined as the lowest concentration that inhibited confluent growth after 2–4 days of incubation on agar plates as described previously for MIC determination of environmental bacteria (87, 134, 135). The MBIC for fungi corresponded to the concentration at which there was no increase in the initial size of the mycelium disk (6 mm). Uncultured media amended with the respective metals served as abiotic controls.

Metal(oid) salt stock solutions were prepared at a concentration of 1 M (Table S5), sterilized through 0.22 µm filters (Pall Co., USA), and stored in the dark at 4°C. The MBIC was defined as the lowest concentration of metal(oid) at which no visible bacterial growth was observed. Visual assessment of bacterial growth, including any changes in colony pigmentation, was recorded after incubation to verify the MIC. Inoculated plates were incubated at 28°C for 48–96 hours in a cell incubator before growth evaluation.

### Scanning transmission electron microscopy and energy-dispersive X-ray analyses

Cultures treated or untreated with the respective heavy metal were collected after 3–7 days of incubation at 30°C. Bacterial cells were then pelleted using low-speed centrifugation. The pellet was fixed with 3% glutaraldehyde prepared in cacodylate buffer at 22°C for 5 minutes and subsequently washed with cold cacodylate buffer. The fixed cells were dehydrated using an ethanol gradient (30%, 50%, 70%, 90%, and 100%), for 5 minutes each. The fixed samples were then applied to a copper mesh coated with holey carbon film 300 (Agar scientific, UK) and immediately examined using a Helios 5 PFIB CXe scanning electron microscope (Thermo Fisher Scientific Brno s.r.o., Czech Republic; acceleration voltage 30 kV, prompt current 0.1 nA) equipped with a STEM 3 + detector operating at either bright field or High-Angle Annular Dark-Field (HAADF), and an EDX spectrometer (30 kV, prompt current 1.6 nA).

## Data Availability

Primary data from sequencing and chemical analysis of samples from the Zlate Hory mining site have been deposited in Zenodo at https://doi.org/10.5281/zenodo.14899081.
